# Fruit and vegetable consumption before and during pregnancy and developmental delays in offspring aged 2 years in Japan

**DOI:** 10.1017/S0007114521002154

**Published:** 2022-04-28

**Authors:** Yudai Yonezawa, Fumihiko Ueno, Taku Obara, Takahiro Yamashita, Mami Ishikuro, Keiko Murakami, Aoi Noda, Tomomi Onuma, Junichi Sugawara, Shigenori Suzuki, Hiroyuki Suganuma, Shinichi Kuriyama

**Affiliations:** 1 Tohoku Medical Megabank Organization, Tohoku University, 2-1 Seiryo-machi, Aoba-ku, Sendai, Miyagi 980-8573, Japan; 2 Innovation Division, KAGOME CO., LTD., 17 Nishitomiyama, Nasushiobara, Tochigi 329-2762, Japan; 3 Division of Molecular Epidemiology, Tohoku University Graduate School of Medicine, 2-1 Seiryo-machi, Aoba-ku, Sendai, Miyagi 980-8575, Japan; 4 Department of Pharmaceutical Sciences, Tohoku University Hospital, 1-1 Seiryo-machi, Aoba-ku, Sendai, Miyagi 980-0872, Japan; 5 Depertment of Gynecology and Obstetrics, Tohoku University Graduate School of Medicine, 2-1 Seiryo-machi, Aoba-ku, Sendai, Miyagi 980-8575, Japan; 6 International Research Institute of Disaster Science, Tohoku University, 468-1 Aramakiaoba, Aoba-ku, Sendai, Miyagi 980-8572, Japan

**Keywords:** Vegetables, Fruit, Child Development, Fetal Development, Pregnancy

## Abstract

The association between fruit and vegetable consumption before and during pregnancy and offspring’s physical growth has been well reported, but no study has focused on offspring’s neurological development. We aimed to explore the association between maternal fruit and vegetable consumption before and during pregnancy and developmental delays in their offspring aged 2 years. Between July 2013 and March 2017, 23 406 women were recruited for the Tohoku Medical Megabank Project Birth and Three-Generation Cohort Study. Fruit and vegetable consumption was calculated using FFQ, and offspring’s developmental delays were evaluated by the Ages & Stages Questionnaires, Third Edition (ASQ-3) for infants aged 2 years. Finally, 10 420 women and 10 543 infants were included in the analysis. Totally, 14·9 % of children had developmental delay when screened using the ASQ-3. Women in the highest quartile of vegetable consumption from pre-pregnancy to early pregnancy and from early to mid-pregnancy had lower odds of offspring’s developmental delays (OR 0·74; 95 % CI 0·63, 0·89 and OR 0·70; 95 % CI 0·59, 0·84, respectively) than women in the lowest quartile. Women in the highest quartile of fruit consumption from early to mid-pregnancy had lower odds of offspring’s developmental delays (OR 0·78; 95 % CI 0·66, 0·92) than women in the lowest quartile. In conclusion, high fruit and vegetable consumption before and during pregnancy was associated with a lower risk of developmental delays in offspring aged 2 years.

Developmental delays in early life including speech and language development, motor development, social-emotional development and cognitive development^(1)^ can affect individuals throughout their life. Generally, developmental delays are determined when a child does not achieve milestones compared with peers of the same age range; the estimated prevalence is approximately 8·4 % in infants under 5 years of age^(2)^. Infants with developmental delays have a higher risk of learning disabilities, behaviour problems and difficulty building friendships later in life^(3)^. They are also at higher risk of educational attainment and well-being compared with children without such disabilities^(4)^ and require lifelong treatment and medical care, resulting in a greater social and economic burden.

Diet during pregnancy has a close relationship with the development of the child. It has been reported that fish, PUFA, folic acid and multivitamin intake before and during pregnancy have a positive effect on the offspring’s neurological development^(5–9)^. Fruit and vegetables are major sources of vitamins and minerals, but the association between fruit and vegetable consumption before and during pregnancy and offspring’s neurological development has not been investigated. Because fruit and vegetable consumption has been reported to be beneficial for children’s physical growth and the prevention of other diseases^(10–13)^, it may also be valuable for children’s neurodevelopment.

Modifying environmental factors before and during pregnancy can prevent developmental disorders and is necessary especially because developmental disorders require long-term treatment and specialised education once it occurs. Identifying the association between dietary habits before and during pregnancy, which is one of the environmental factors within one’s control, and offspring’s developmental delays may provide valuable clues to aid in its prevention. Therefore, this study aimed to investigate the association between fruit and vegetable consumption before and during pregnancy and offspring’s development at 2 years of age, when they are at the early stages of development. In addition, we investigated the association between changes in fruit and vegetable consumption before and during pregnancy and offspring’s development at 2 years of age to evaluate the appropriate period for these consumption.

## Methods

### Study design and population

**Fig. 1.**
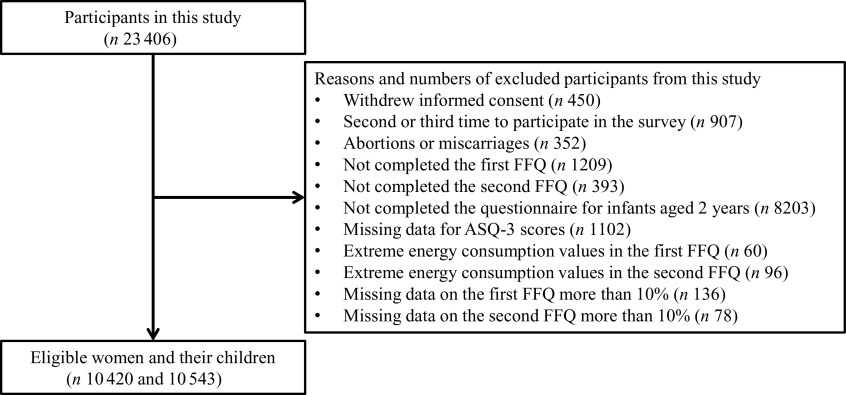
Flow chart of participant exclusion criteria in this study. This flow chart describes the exclusion criteria and the number of participants, excluded participants, and eligible participants in total. ASQ-3, Ages & Stages Questionnaires, Third Edition.

This study was based on data obtained in the Tohoku Medical Megabank Project Birth and Three-Generation Cohort Study (TMM BirThree Cohort Study). The TMM BirThree Cohort Study was a prospective cohort study undertaken in Miyagi Prefecture, Japan, and the design and methods have been reported previously^(14,15)^. Between July 2013 and March 2017, 23 406 pregnant women, including women who participated in the study multiple times for different pregnancies, were recruited. Written informed consent was obtained from all participants. We used the data set as of 1 April 2020. The flow chart of participant exclusion criteria is shown in Fig. 1. Among women who participated in the study multiple times for different pregnancies, data from the second and subsequent participation were excluded to eliminate the effect of repeated measurements on a single participant. This study was conducted according to the guidelines stipulated in the Declaration of Helsinki, and all procedures involving human participants were approved by the institutional review board of the Tohoku University Tohoku Medical Megabank Organization (2019-4-057) and KAGOME CO., LTD. (2018-R10).

### Dietary assessment

Two semi-quantitative FFQ were used to evaluate dietary consumption during two different periods. The first FFQ that was administered in early pregnancy enquired about the frequency and amount of food and beverages consumed in the past year to evaluate dietary consumption from pre- to early pregnancy. The second FFQ was administered at mid-pregnancy and enquired about the frequency and amount of food and beverages consumed since the first FFQ was completed to evaluate dietary consumption from early to mid-pregnancy. Mean response periods of the first and second FFQ in the participants included in the analysis were 19·5 (sd 7·2) weeks and 27·7 (sd 5·5) weeks of gestation, respectively. Frequency and amount of 130 food items and beverage consumption were evaluated through each FFQ. Frequency and amount of consumption of food items and beverages were converted into average daily consumption by multiplying the frequency and quantity. These items included eighteen fruits, twenty-six vegetables, eighteen meats, nineteen fish, nine grains, four potatoes, seven beans and seven dairy products. The total consumption of each dietary group was identified by summing the responses to the consumption query of each dietary group. Total energy and nutrient consumption such as carotene, vitamin C, vitamin K and folate were calculated using the Standard Tables of Food Composition in Japan (Fifth Revised and Enlarged Edition 2005)^(16)^. The consumption of each dietary and nutrient was energy-adjusted using the residual method^(17)^, and the values were included in the analysis as continuous or categorical values, which were divided into quartiles. Spearman’s correlation coefficient between fruit consumption from pre- to early pregnancy and that from early to mid-pregnancy was 0·559 (online Supplementary Table S1). Similarly, Spearman’s correlation coefficient between vegetable consumption from pre- to early pregnancy and that from early to mid-pregnancy was 0·600. Additionally, we defined four categories based on changes from pre- to mid-pregnancy in the total fruit, vegetable, meat, fish, grain, potato, bean, dairy product, carotene, vitamin C, vitamin K and folate consumption. The definitions of the four categories were described as follows: women whose answers to the first and second FFQ were in the first or second quartile (low-low group), women whose answers to the first FFQ were in the first or second quartile and whose answers to the second FFQ were in the third or fourth quartile (low-high group), women whose answers to the first FFQ were in the third or fourth quartile and whose answers to the second FFQ were in the first or second quartile (high-low group), and women whose answers to the first and second FFQ were in both the third or fourth quartile (high-high group). More detailed methods are described elsewhere^(10)^.

### Outcome variables

The Ages & Stages Questionnaires, Third Edition (ASQ-3) was used to evaluate offspring’s developmental delays. The ASQ-3 is a broadband questionnaire to screen offspring’s developmental delays, and it consists of twenty-one age-specific questionnaires for ages 1–66 months. In the present study, we used the validated Japanese translation of the ASQ-3^(18)^ and obtained information using the ASQ-3 for infants aged 2 years. Each questionnaire contains thirty questions divided into five developmental domains: ‘communication’, ‘fine motor’, ‘gross motor’, ‘problem solving’ and ‘personal-social’. Each domain has a set of six items, and each item is scored as 10, 5 and 0 corresponding to ‘yes’, ‘sometimes’ and ‘not yet’, respectively. The total score ranges from 0 to 60 for each domain. Each domain was classified as a failed domain when the score was <2 sd below the mean^(19)^. Infants were identified as having developmental delays if they failed in one or more of the domains^(19)^.

### Confounders

Variables related to birth dates of mothers, fathers and their infants were obtained from medical records and the informed consent form. Maternal age (years) and paternal age at birth of their infant (years) were calculated by subtracting the infants’ birth dates from each of their parents’ birth dates. Infant birth season was based on the birth date of infants categorised under any one of the four seasons, ‘spring (March–May)’, ‘summer (June–August)’, ‘autumn (September–November)’ and ‘winter (December–February)’. Variables related to maternal height (cm), pre-pregnancy weight (kg), parity (never, one or more) and infant sex (male and female) were obtained from medical records. Maternal age was divided into four categories (<25, 25–29, 30–34 and ≥35 years), and paternal age was divided into six categories (<25, 25–29, 30–34, 35–39, 40–44 and ≥45 years). Pre-pregnancy BMI (kg/m^2^) was calculated by dividing the pre-pregnancy weight (kg) by the square of maternal height (m^2^) and was classified into three categories (<18·5, 18·5–24·9 and ≥25·0 kg/m^2^). Variables related to cigarette smoking (never, stopped before pregnancy, stopped after pregnancy and current), alcohol consumption (never, former and current), folic acid supplementation during early pregnancy (yes and no) and fertility treatment (yes and no) were obtained from the questionnaire during early pregnancy. Data on variables related to household income (<4 000 000, 4 000 000–5 999 999 and ≥6 000 000 Japanese Yen/year) were obtained from the questionnaire during mid-pregnancy. Maternal educational attainment data (high school graduate or less, junior college or vocational college graduate, university graduate or above, others), maternal personal pervasive developmental disorders history (yes and no) and paternal personal pervasive developmental disorders history (yes and no) were gathered a year after birth. Breast-feeding duration data were obtained from the questionnaire for infants aged 6 months and 1 year and were classified into two categories (<6 and ≥6 months). Frequencies of fruit, green vegetable, red and yellow vegetables, and light-coloured vegetable consumption in the offspring at age 2 years in the past week (never, once or twice a week, three or four times a week, five or six times a week, once a day, twice a day, three or more times a day) were obtained from the questionnaire for infants aged 2 years. Total meat, fish, grain, potato, bean and dairy product consumption and their changes from pre- to mid-pregnancy were also included in the analyses as confounders.

### Statistical analysis

Continuous variables were expressed as mean and standard deviation, and categorical variables were indicated as frequencies and percentages. The association between dietary and nutrient consumption and offspring’s developmental delays was evaluated by multivariate logistic regression analysis to calculate the OR, 95 % CI and *P* for trend. The association between changes in dietary and nutrient consumption and offspring’s developmental delays was evaluated by the same method used to calculate the OR, 95 % CI. Missing data for confounders were imputed using multivariate imputation methods by chained equations assuming missing data at random^(20)^. For the present study, multivariate imputation methods by chained equations imputed each incomplete confounder by generating plausible synthetic values based on exposure, outcome and the other confounders in the data. We independently analysed twenty copies of the data, each with missing values imputed in the multivariate analyses. The first quartile and low-low group were used as the reference group. In a sub-analysis, OR, 95 % CI and *P* for trend were estimated using multivariate logistic regression with generalised estimating equations, specifying an exchangeable correlation structure. Generalised estimating equations were used to account for the correlation of repeated observations (multiple births, multiple participation) by a single participant. Spearman’s correlation analysis was used to assess correlations among mothers and their offspring’s fruit and vegetable consumption. *P* < 0·05 was considered statistically significant. Statistical analyses were performed with R, version 4.0.2.

## Results

### Characteristics of the study population

In total, 10 420 women and 10 543 infants (including twins and triplets) were included in the analysis (Fig. 1). Among the women included in the analysis, the mean age was 32·1 (sd 4·8) years old, the mean maternal height was 158·5 (sd 5·4) cm and the mean pre-pregnancy weight was 53·9 (sd 9·1) kg (Table 1). A small number of mothers and fathers had personal pervasive developmental disorders history, and approximately 10 % parents underwent fertility treatment. Totally, 1572 infants (14·9 %) had developmental delay when screened using the ASQ-3. Comparison of carotene, vitamin C and folate consumption between fruit and vegetable quartiles showed that the fourth quartile of vegetable consumption had higher carotene and folate consumption than the fourth quartile of fruit consumption (online Supplementary Table S2–S5). Vitamin C consumption was slightly higher in the fourth quartile of fruit consumption than the fourth quartile of vegetable consumption. No strong correlation of fruit and vegetable consumption before and during pregnancy with the frequency of fruit and vegetable consumption by the offspring at age 2 years was observed (online Supplementary Table S1). The information on the frequency of dietary consumption in offspring aged 2 years is shown in Supplementary Tables S6–S9.

**Table 1.** Characteristics of the study population (Numbers and percentages; mean values and standard deviations)

	*n*	%
*n*	10 543	
Maternal characteristics		
Age (years)		
Mean	32·1	
sd	4·8	
Height (cm)		
Mean	158·5	
sd	5·4	
Pre-pregnancy weight (kg)		
Mean	53·9	
sd	9·1	
Pre-pregnancy BMI*		
<18·5 (kg/m^2^)	1453	13·8
18·5–24·9 (kg/m^2^)	7710	73·1
≥25·0 (kg/m^2^)	1224	11·6
Missing	156	1·5
Parity		
Never	4802	45·5
One or more	5722	54·3
Missing	19	0·2
Educational attainment		
High school graduate or less	2901	27·5
Junior college or vocational college graduate	3703	35·1
University graduate or above	2809	26·6
Others	22	0·2
Missing	1108	10·5
Household income		
<4 000 000 (Japanese Yen/year)	3443	32·7
4 000 000–5 999 999 (Japanese Yen/year)	3409	32·3
≥6 000 000 (Japanese Yen/year)	3287	31·2
Missing	404	3·8
Cigarette smoking		
Never	6690	63·5
Stopped before pregnancy	2496	23·7
Stopped after pregnancy	1167	11·1
Current	162	1·5
Missing	28	0·3
Alcohol consumption		
Never	4774	45·3
Former	3592	34·1
Current	2151	20·4
Missing	26	0·2
Folic acid supplementation during pregnancy		
Yes	6265	59·4
No	4265	40·5
Missing	13	0·1
Personal PDD history		
Yes	4	0·0
No	9492	90·0
Missing	1047	9·9
Fertility treatment		
Yes	1238	11·7
No	9280	88·0
Missing	25	0·2
HG from conception to early pregnancy		
No	1483	14·1
Just nausea	4793	45·5
Vomiting, but was able to eat	3197	30·3
Vomiting, and was unable to eat	1055	10·0
Missing	15	0·1
HG from early to mid-pregnancy		
No	7039	66·8
Just nausea	2414	22·9
Vomiting, but was able to eat	985	9·3
Vomiting, and was unable to eat	85	0·8
Missing	20	0·2
Paternal characteristics		
Personal PDD history		
Yes	5	0·0
No	9491	90·0
Missing	1047	9·9
Infant characteristics		
Infant sex		
Male	5468	51·9
Female	5065	48·0
Missing	10	0·1
Birth season		
Spring (March–May)	2655	25·2
Summer (June–August)	2516	23·9
Autumn (September–November)	2776	26·3
Winter (December–February)	2596	24·6
Breast-feeding duration		
<6 (months)	1864	17·7
≥6 (months)	7056	66·9
Missing	1623	15·4
ASQ-3		
Failed one domain	1004	9·5
Failed two domains	284	2·7
Failed three domains	154	1·5
Failed four domains	62	0·6
Failed five domains	68	0·6
Failed one or more of the domains	1572	14·9

ASQ-3, Ages & Stages Questionnaires, Third Edition; HG, hyperemesis gravidarum; PDD, pervasive developmental disorder.

* Pre-pregnancy BMI was calculated by dividing the pre-pregnancy weight (kg) by the square of maternal height (m^2^).

### Cut-off scores of each Ages & Stages Questionnaires, Third Edition domain and number of infants below the cut-off score

Cut-off scores of each ASQ-3 domain and number of infants below the cut-off score are shown in Table 2. The cut-off score of communication was lowest and that of fine motor was the highest. The percentage of infants with problem-solving delays was the lowest (4·2 %), while gross motor delays were the highest (5·8 %).

**Table 2.** Cut-off scores of each Ages & Stages Questionnaires, Third Edition (ASQ-3) domain and the number of infants below the cut-off score (Numbers and percentages)

	Cut-off scores*	Number of infants below the cut-off score	
		*n*	%
Communication	18·1	548	5·2
Gross motor	35·8	612	5·8
Fine motor	36·5	455	4·3
Problem solving	28·6	438	4·2
Personal social	30·9	569	5·4

* Each domain was classified as a failed domain when the score was 2 sd below the mean.

### Energy-adjusted fruit and vegetable consumption from pre- to mid-pregnancy and offspring’s developmental delays

In the multivariable models, women in the highest quartile of fruit consumption from early to mid-pregnancy had lower odds of offspring’s developmental delays (OR 0·78; 95 % CI 0·66, 0·92) than women in the lowest quartile (Table 3). In addition, high fruit consumption from early to mid-pregnancy was negatively associated with odds of offspring’s developmental delays (*P* = 0·004). Women in the highest quartile of vegetable consumption from pre-pregnancy to early pregnancy and from early to mid-pregnancy had lower odds of offspring’s developmental delays (OR 0·74; 95 % CI 0·63, 0·89 and OR 0·70; 95 % CI 0·59, 0·84, respectively) than women in the lowest quartile. High vegetable consumption from pre-pregnancy to early pregnancy and from early to mid-pregnancy was negatively associated with odds of offspring’s developmental delays (*P* = 0·004 and *P* < 0·001, respectively). The same results were also obtained in the analysis using GEE (online Supplementary Table S10). In addition, by analysing both of these periods of vegetable consumption in the same model, only vegetable consumption from early to mid-pregnancy was negatively associated with odds of offspring’s developmental delays (*P* = 0·014) (online Supplementary Table S11). The effect size of vegetable consumption on ASQ-3 score was greater than that of fruit consumption in both periods. Moreover, higher carotene, vitamin C and folate consumption from pre-pregnancy to early pregnancy and these from early to mid-pregnancy were negatively associated with odds of offspring’s developmental delays (online Supplementary Table S12).

**Table 3.** Energy-adjusted fruit and vegetable consumption from pre- to mid-pregnancy and offspring’s developmental delays* (Odd ratios and 95 % confidence intervals)

	From pre- to early pregnancy					From early to mid-pregnancy				
Quartiles	Daily consumption	Crude		Multivariable model†		Daily consumption	Crude		Multivariable model†	
		OR	95 % CI	OR	95 % CI		OR	95 % CI	OR	95 % CI
			lower, upper		lower, upper			lower, upper		lower, upper
Fruit										
First quartile	< 76·2 g/d	Reference		Reference		< 73·8 g/d	Reference		Reference	
Second quartile	76·3–130·4 g/d	0·94	0·81, 1·10	0·90	0·77, 1·06	73·8– 124·5 g/d	1·10	0·95, 1·28	1·09	0·93, 1·27
Third quartile	130·4–199·9 g/d	1·00	0·86, 1·16	1·01	0·86, 1·19	124·6–191·8 g/d	0·99	0·85, 1·15	1·01	0·86, 1·18
Fourth quartile	≥ 199·9 g/d	0·84	0·72, 0·98	0·86	0·73, 1·01	≥ 191·8 g/d	0·75	0·64, 0·88	0·78	0·66, 0·92
* P* for trend		0·07		0·28			< 0·001		0·004	
Vegetables										
First quartile	< 100·1 g/d	Reference		Reference		< 95·6 g/d	Reference		Reference	
Second quartile	100·1–139·3 g/d	0·77	0·67, 0·89	0·75	0·64, 0·87	95·7–131·4 g/d	0·91	0·79, 1·05	0·88	0·76, 1·03
Third quartile	139·4–190·2 g/d	0·75	0·65, 0·88	0·77	0·65, 0·91	131·4–179·4 g/d	0·83	0·72, 0·96	0·83	0·71, 0·98
Fourth quartile	≥ 190·3 g/d	0·70	0·61, 0·82	0·74	0·63, 0·89	≥ 179·4 g/d	0·67	0·58, 0·79	0·70	0·59, 0·84
* P* for trend		< 0·001		0·004			< 0·001		< 0·001	

HG, hyperemesis gravidarum; PDD, pervasive developmental disorder.

* The odds of offspring developmental delays were evaluated using ASQ-3.

† Adjusted for maternal age (<25, 25–29, 30–34 and ≥35 years), pre-pregnancy BMI (<18·5; 18·5–24·9 and ≥25·0 kg/m^2^), parity (never; one or more), educational attainment (high school graduate or less; junior college or vocational college graduate; university graduate or above; others), household income (<4 000 000; 4 000 000–5 999 999; ≥6 000 000 Japanese Yen/year), cigarette smoking (never; stopped before pregnancy; stopped after pregnancy; current), alcohol drinking (never; former; current), folic acid supplementation during pregnancy (yes; no), maternal PDD history (yes; no), fertility treatment (yes; no), HG from conception to early pregnancy or HG from early to mid-pregnancy (no; just nausea; vomiting, but was able to eat; vomiting, and was unable to eat), paternal PDD history (yes; no), infant sex (male; female), birth season (spring; summer; autumn; winter), breast-feeding duration (<6, ≥6 months), frequency of fruit, green vegetable, red and yellow vegetable and light-coloured vegetable consumption in the offspring at age 2 years (never; once or twice a week; three or four times a week; five or six a week; once a day; twice a day; three or more times a day) and total fruit, vegetable, meat, fish, grain, potato, bean, and daily product consumption (in quartiles) except for exposures.

### Changes in fruit and vegetable consumption from pre- to mid-pregnancy and offspring’s developmental delays

No significant differences were identified between changes in fruit consumption from pre- to mid-pregnancy and offspring’s developmental delays (Table 4). Compared with women with low vegetable consumption before and during pregnancy (low-low group), women with high consumption only from early to mid-pregnancy (low-high group) and women with high consumption before and during pregnancy (high-high group) had offspring with lower odds of developmental delays (OR 0·84; 95 % CI 0·70, 1·00 and OR 0·82; 95 % CI 0·71, 0·95, respectively). No association was identified in women with high consumption only from pre- to early pregnancy (high-low group) (OR 0·92; 95 % CI 0·77, 1·10). Similar results were also observed in the analysis using GEE (online Supplementary Table S13). Moreover, women with high carotene, vitamin C and folate consumption before and during pregnancy (high-high group) had offspring with lower odds of developmental delays than women with low consumption before and during pregnancy (low-low group) (online Supplementary Table S14).

**Table 4.** Changes in fruit and vegetable consumption from pre- to mid-pregnancy and offspring developmental delays* (Odd ratios and 95 % confidence intervals)

Quartiles		Crude		Multivariable model†	
		OR	95 % CI	OR	95 % CI
	*n*		Lower, upper		Lower, upper
Fruit					
Low-low‡	3732	Reference		Reference	
Low-high§	1539	0·86	0·73, 1·02	0·87	0·73, 1·04
High-low||	1539	1·08	0·92, 1·26	1·10	0·93, 1·30
High-high¶	3733	0·84	0·74, 0·96	0·88	0·77, 1·01
Vegetables					
Low-low‡	3845	Reference		Reference	
Low-high§	1426	0·81	0·68, 0·96	0·84	0·70, 1·00
High-low||	1426	0·88	0·75, 1·04	0·92	0·77, 1·10
High-high¶	3846	0·74	0·66, 0·84	0·82	0·71, 0·95

ASQ-3, Ages & Stages Questionnaires, Third Edition; HG, hyperemesis gravidarum; PDD, pervasive developmental disorder.

* The odds of offspring developmental delays were evaluated using ASQ-3.

† Adjusted for maternal age (<25; 25–29; 30–34; ≥35 years), pre-pregnancy BMI (<18·5; 18·5–24·9; ≥25·0 kg/m^2^), parity (never; one or more), educational attainment (high school graduate or less; junior college or vocational college graduate; university graduate or above; others), household income (<4 000 000; 4 000 000–5 999 999; ≥6 000 000 Japanese Yen/year), cigarette smoking (never; stop before pregnancy; stop after pregnancy; current), alcohol drinking (never; former; current), folic acid supplementation during pregnancy (yes; no), maternal PDD history (yes; no), fertility treatment (yes; no), HG from conception to early pregnancy or HG from early to mid-pregnancy (no; just nausea; vomiting, but was able to eat; vomiting, and was unable to eat), paternal PDD history (yes; no), infant sex (male; female), birth season (spring; summer; autumn; winter), breast-feeding duration (<6, ≥6 months), frequency of fruit, green vegetable, red and yellow vegetable and light-coloured vegetable consumption in the offspring at age 2 years (never; once or twice a week; three or four times a week; five or six a week; once a day; twice a day; three or more times a day) and changes in fruit, vegetable, meat, fish, grain, potato, bean, and daily product consumption (low-low; low-high; high-low; high-high) except for exposures.

‡ Women whose answers to the first and second FFQs were in the first or second quartile.

§ Women whose answers to the first FFQ were in the first or second quartile and whose answers to the second FFQ were in the third or fourth quartile.

|| Women whose answers to the first FFQ were in the third or fourth quartile and whose answers to the second FFQ were in the first or second quartile.

¶ Women whose answers to the first and second FFQ were in both the third or fourth quartile.

The associations between fruit and vegetable consumption and each domain of ASQ-3 are presented in online Supplementary Tables S15 and S16.

## Discussion

This study investigated the association between fruit and vegetable consumption before and during pregnancy and development of offspring aged 2 years. The results showed that high fruit and vegetable consumption before and during pregnancy was associated with a lower risk of developmental delays in offspring, aged 2 years.

Fruit and vegetables are rich sources of various vitamins, minerals, carotenoids and phenolics^(21)^. Previous studies have shown that folic acid and/or multivitamin intake before and during pregnancy were beneficial for offspring’s development^(7–9)^. Fruit and vegetables abundantly contain pro-vitamin A, vitamin C, vitamin K and folate^(21,22)^, and these three components are most abundantly consumed from vegetables in Japan^(23)^. In the results of the present study, an inverse association between higher consumption of carotene (precursor to vitamin A), vitamin C and folate and offspring’s developmental delays was observed (online Supplementary Table S12). Vitamin A supplementation during pregnancy has been reported to be potentially beneficial in reducing maternal anaemia and maternal infections in vitamin A-deficient areas^(24)^. Another previous study reported that vitamin C may benefit pregnancies with intra-uterine growth retardation and preeclampsia^(25)^. Vitamin C is also involved in the absorption of Fe in the body and therefore may have a useful effect during pregnancy by preventing anaemia. Folate is a methyl donor necessary for DNA synthesis and cell division, and the value of folate for the prevention of neural tube defects is well known^(26,27)^. In fact, anaemia, infections, intra-uterine growth retardation and preeclampsia during pregnancy were reported to be risk factors for offspring’s development^(28–32)^. Therefore, carotene, vitamin C and folate consumption could partly explain the association between fruit and vegetable consumption and offspring’s developmental delays.

Our results showed that vegetable consumption may have a greater influence on offspring’s development than fruit consumption. Vegetables accounted for 53·1 %, 40·0 % and 38·0 % of the dietary vitamin A, vitamin C and folic acid intake, respectively, in Japan^(23)^. Fruit accounted for 5·4, 31·9 and 5·9 % of the dietary vitamin A, vitamin C and folic acid intake, respectively, in Japan^(23)^. In our sub-analyses, the fourth quartile of vegetable consumption had higher carotene and folate consumption than the fourth quartile of fruit consumption in both first and second FFQ (online Supplementary Tables S4 and S5). The fact that vegetable consumption had a greater influence on dietary vitamin intake than fruit consumption may be one reason for vegetable consumption having a greater effect on offspring’s development than fruit consumption.

This study is the first to investigate the relationship between fruit and vegetable consumption, the timing of this consumption and the offspring’s developmental delays. Several previous studies reported that folic acid intake before and during pregnancy reduces offspring’s developmental delays^(7,8)^. Pre-pregnancy folic acid is essential for the development of the central nervous system which begins immediately after conception; therefore, folic acid intake prior to pregnancy is critical^(33)^. However, our results showed that offspring’s developmental delays were lower not only with higher fruit and vegetable consumption before and throughout pregnancy but also with higher consumption from early pregnancy. These results may suggest that fruit and vegetable consumption not only before and during early pregnancy but also throughout pregnancy may play an important role in the development of the central nervous system. The knowledge on the timing of certain food and nutritional component consumption is still limited. Therefore, further research is necessary to provide guidance on appropriate dietary timing and to elucidate the underlying mechanisms.

In addition to fruit, vegetables and the vitamins contained in them, several previous studies reported that fish and PUFA such as DHA and EPA were beneficial for neurodevelopment in offspring^(5,6)^. Comparing fish and unsaturated fatty acid consumption by quartiles of fruit and vegetable consumption, the higher the quartile of vegetable consumption, the higher the fish and unsaturated fatty acid consumption (online Supplementary Tables S2–S5). The difference in fish consumption among the quartiles of vegetable consumption was a maximum of 13·7 g/d (online Supplementary Table S4). However, this difference in consumption is not expected to influence the results of the present analysis because we adjusted for fish consumption as a confounder.

As the result confirming the correlation of the fruit and vegetable consumption before and during pregnancy with the frequency of fruit and vegetable consumption by the offspring aged 2 years showed, no strong correlation was found (online Supplementary Table S1). This suggests that the offspring of mothers who had higher fruit and vegetables did not necessarily consume fruit and vegetables. In other words, the results of this study do not suggest that the amount of fruit and vegetables consumed by the offspring themselves influenced their developmental delay.

This study has several limitations. First, ASQ-3 is a screening tool, not a diagnostic tool, for developmental delays. However, ASQ-3 is considered to have high reliability because it has been validated in many countries around the world and used in a variety of studies^(18,34,35)^. Second, in the present study, the results of ASQ-3 were used only at 2 years of age and it is necessary to study the offspring at later ages as the follow-up study progresses. Third, the first and second FFQ were made by modifying the FFQ used in the Japan Public Health Centre-Based Prospective study^(36,37)^ and were added to the response option ‘constitutionally unable to eat it’ to identify genetic factors. The FFQ used in the Japan Public Health Centre-Based Prospective study was validated in the general Japanese population^(36–39)^, but not in pregnant women. Fourth, it was not possible to clarify the causal relationship between exposures and outcomes because this was an observational study. Fifth, although Fe intake during pregnancy is known to be associated with cognitive function in the offspring, Fe intake during pregnancy could not be taken into account in this study.

The present study had several strengths. The TMM BirThree Cohort Study is a prospective birth cohort design with a large sample size. It was possible to evaluate changes in fruit and vegetable consumption before and during pregnancy and find the association with offspring’s development. Moreover, we could research the long-term effect of fruit and vegetable consumption before and during pregnancy using two kinds of FFQ given at different periods.

In conclusion, fruit and vegetable consumption before and during pregnancy was associated with a lower risk of developmental delays in offspring aged 2 years. Dietary habits before and during pregnancy might be one of the key factors that influence the growth of offspring.

## Supplementary material

For supplementary material accompanying this paper visit https://doi.org/10.1017/S0007114521002154. click here to view supplementary material
